# Editorial for the Special Issue “Food and Microbial Bioprocesses”

**DOI:** 10.3390/microorganisms12040736

**Published:** 2024-04-03

**Authors:** Humberto Hernández-Sánchez, Gustavo F. Gutiérrez-López

**Affiliations:** Departamento de Ingeniería Bioquímica, Instituto Politécnico Nacional, Escuela Nacional de Ciencias Biológicas, Carpio y Plan de Ayala S/N, Santo Tomás, Mexico City C.P. 11340, Mexico; hhernan1955@gmail.com

The aim of this Special Issue was to provide readers with a holistic, systematic, and integrative approach to microbial processes involved in the production of selected foods, nutraceuticals, and bioactive materials by using modern biotechnological tools. The production of such commodities is receiving a great deal of attention, given their growing demand due to their important health benefits. The preparation of these goods, is, in essence, an innovative activity in which scientific and technological tools, such as genetic engineering, molecular biology, micro- and nano-technology, morphostructural engineering, and modern scale-up and scale-down procedures, amongst others, converge and interact with common goals as, for example, on the application of bioprocesses for the identification, production, and recovery of bioproducts as functional foods. The approach is to supply enough healthy food and bioproducts for an growing and demanding population. In this respect, sequential and integrative approaches to manufacturing must be undertaken, while considering the bioproduct at the centre of the attention, as in the production of nutraceuticals and functional foods, which can be accomplished by means of bioprocessing, including upstream and downstream operations, in which a great number of microorganisms and production methods may be involved, and are the key constituents of the production task. Also, it must be borne in mind that one of the first steps in the production stage is knowledge of the matrixes that constitute the bioproducts and their relations with microbial kinetics and, it is precisely in this area that this Special Issue was conceived (Figure 1), which consists of state-of-the-art original contributing articles covering key aspects of the relations of microorganisms with food-related materials and bioprocesses, which were as follows. First, the production of ROS and RNS in macrophages activated by the interaction of probiotic bacteria and *Escherichia coli* pathotypes [1]. Such activation was revealed through flow cytometry, and it was observed that the detected effects depended on the kind of probiotic used and on the strain of *E. coli* used. The effect of agave fructans on chemical and morphometric characteristics of kefir grains [2]. The amount of inoculum and fermentation at 25 °C were the best for production, and it was noted that the addition of agave fructans in the medium promoted the production of biomass with respect to the lactose-free culture medium. The survival of a *Lactobacillus fermentum* in the production of high-oleic palm oil (HOPO) macroemulsion [3]. It was found that the amount of HOPO influenced the survival of bacteria, and the spray-drying process showed a similar viable cell count before and after spray drying. The brewing process of a craft pale ale by using a non-*Saccharomyces* yeast as a starter [4]. It was observed that no mycotoxins, arsenic, lead, methanol, or microbiological spoilage were found, and it was noteworthy that, in a sensory test, this beer was preferred to a Belgian-style pale ale prepared by using *S. cerevisiae*, and the physicochemical, the final ethanol concentration and other features fulfilled national and international standards. The biodegradation and bioconversion of edible mushrooms [5]. It was observed that, *Pleurotus eryngii* was the best option for scaling up procedures among the various exotic mushrooms tested, including *Lentinula edodes*, and the analytical results provided original knowledge on high-scale mushroom-production processes. The screening of probiotic bacteria from goat’s milk [6]. The authors found that three of the isolated strains had an auto-aggregative phenotype and produced bacteriocins with good heat stability. The probiotic potential of the strains included antibacterial activity, bile tolerance and resistance to simulated GI digestion, good adhesion and, in general, were safe. The identification of bacterial communities from fermented soybeans (FSBs) from different ethnical groups [7]. The results showed antagonistic behaviour of *Bacillus* against some pathogens, and the presence of *Bacillus* in all FSBs and *Vagococcus* in the Shan FSB suggested that these may possess beneficial bacteria. The use of probiotics for improving small ruminant meat [8]. The authors reported that benefits were related with applications of probiotics. The results from the revision did not show consistent findings, thus the mechanisms behind the successful production of quality meat are not clear, and are possibly due to the usage of diverse probiotic bacteria, the dosage used, time of application, breed, age, and health conditions of the ruminants. The effect of freezing on the survival rate of encapsulated probiotic *Lactobacillus acidophilus* LA5 by using a novel flash freeze drying process was also reported [9]. In this work, the use of flash 13 freeze-drying (FFD), a cyclic and rapid increasing and decreasing of pressure during primary drying was studied, obtaining total process times of about a third that of the classical freeze-drying. The above articles provide a holistic perspective of the complex relationships between food production and microbial kinetics, with a specific focus on human wellbeing, and the collection of articles in this Special Issue provides a good example of the current state of the relations between food science, technology, and biotechnology. Future works should be directed, amongst other topics, to studying the fundamental molecular aspects that may control the different responses depicted in this Special Issue and based, for instance, on a systematic biological approach and, on the other hand, to investigate modern scale-up procedures for complex production schemes. Also, studies based on green technologies and the circular economy should be promptly addressed. We hope that readers will find in this collection of works in the form articles around the common goal of relating food and microbial bioprocesses, to be an organized and balanced contribution to the field.

## Figures and Tables

**Figure 1:**
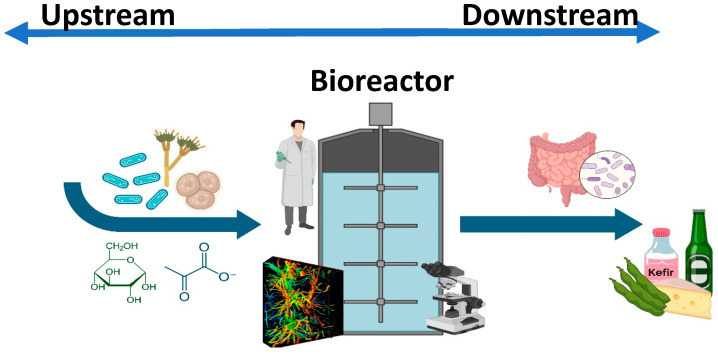
Overall representation of the production of foods by bioprocessing. The figure depicts both upstream and downstream processing, and includes, as examples: microbial inoculum, the nutrients included in the medium, a bioreactor with a microorganism such as a fungus growing inside, and a few examples of final products obtained by bioprocessing, such as kefir, beer, fermented vegetables and cheese, and their passage throughout the gastrointestinal tract.
